# Co‐creating an Indigenous‐led virtual health services model for Indigenous Australians living with chronic disease

**DOI:** 10.1111/ajr.13206

**Published:** 2024-12-16

**Authors:** Bushra Farah Nasir, William MacAskill, Floyd Leedie, Priya Martin, Khorshed Alam, Katharine Wallis, Matthew McGrail, Srinivas Kondalsamy‐Chennakesavan

**Affiliations:** ^1^ Rural Clinical School, Faculty of Medicine The University of Queensland Toowoomba Queensland Australia; ^2^ Griffith University Rural Clinical School Toowoomba Queensland Australia; ^3^ Rural Medical Education Australia Toowoomba Queensland Australia; ^4^ Goondir Health Services Dalby Queensland Australia; ^5^ School of Business, and Centre for Health Research University of Southern Queensland Toowoomba Queensland Australia; ^6^ General Practice Clinical Unit, Medical School The University of Queensland Brisbane Queensland Australia; ^7^ Rural Clinical School, Faculty of Medicine The University of Queensland Rockhampton Queensland Australia

**Keywords:** digital health, Indigenous health, rural health, telehealth, virtual health services

## Abstract

**Objective:**

To describe the co‐design process and understand consumer perspectives of a virtual health services (VHS) model of primary healthcare delivery, for Indigenous Australians with chronic disease and living in regional, rural, and remote Queensland.

**Design:**

Using decolonising methodologies, the study used an Indigenous consensus method to undertake the co‐design process and generate findings. For analysis, a qualitative interpretive‐description framework was applied. Thematic analysis generated themes, describing consumer perspectives of virtual healthcare models.

**Setting:**

The Goondir Health Services (Aboriginal Community Controlled Health Organisation) operating clinics in rural and remote Queensland.

**Participants:**

Fourteen Indigenous VHS consumers who resided in Modified Monash Model 3–7 communities across Queensland, met the eligibility criteria and provided informed consent.

**Results:**

Two themes emerged: (1) personalised approaches to autonomous care using digital technologies, with two sub‐themes of the benefits and challenges of technology, and the integration of culturally inclusive healthcare elements; (2) person‐centred, culturally appropriate healthcare elements within a VHS model, with three sub‐themes on the vital role of health coaches, the importance of community connections, and enabling holistic personalised healthcare access.

**Conclusion:**

This study provides important consumer perspectives on the potential of VHS models of health care to empower Indigenous healthcare service consumers. VHS holds promise on multiple fronts: improved access, timeliness, continuity of care, and culturally sensitive health care, enabling improved self‐management of chronic conditions, and provide crucial support from local Indigenous healthcare providers within the community. Future research on the sustainability and impact of personalised, consumer‐centric digital health services in Indigenous populations is essential.

**What is already known on this subject?**

- Virtual health services provide opportunities to bridge healthcare access gaps, especially for under‐serviced or priority populations.
- The use of virtual health services, such as telehealth, has demonstrated cultural acceptability and value for Indigenous Australian populations.
- There is a lack of evidence on Indigenous‐led and designed virtual health services integrating digital health technologies for rural Indigenous populations.

**What this paper adds?**

- The co‐design of an Indigenous‐led virtual health service model provides an innovative approach to creating digital primary health care models.
- A virtual health service should prioritise a consumer‐centred model, through establishing consumer autonomy and integrating culturally appropriate elements.
- Improving access to healthcare by enabling digital technology that meets consumer‐identified needs, are important when developing virtual health service models for Indigenous consumers.

## 1 INTRODUCTION

Virtual healthcare services (VHS) are increasingly being used to bridge healthcare access gaps across the world. VHS systems provide consumers the ability to connect remotely with healthcare service providers using digital technology.^1^ Examples of VHS include telehealth, mobile health apps and messaging, and remote patient monitoring (RPM).^2^ Such models of care can increase healthcare capacity by reducing costs, saving time for both patients and providers, and minimising the need for additional physical infrastructure.^3^ In recent years, digital primary healthcare (PHC) models based on VHS have rapidly transformed the delivery of health services, partly driven by the COVID‐19 pandemic.^1^, ^4^

Australia, like many other countries, has also fast‐tracked its digital health delivery capabilities to ensure efficient and prompt service delivery during the pandemic.^4^ Vast geographical distances in Australia have led to the facilitation of and access to quality PHC services using virtual models of care.^5^ For underserved and priority populations, such as Indigenous populations and those living in rural or remote regions, the use of VHS models can provide an innovative approach to bridge healthcare access gaps, modernise efficient service delivery and improve patient health outcomes.^5^, ^6^ The use of digital technology, such as telehealth, for Indigenous Australians has shown to be a viable strategy to bridge healthcare access barriers.^6^, ^7^, ^8^, ^9^ Transforming health care through digital innovation, beyond telehealth services, can also meet the increasing demand and capacity for PHC services in under‐serviced areas.^10^

### 1.1 Virtual models of health care for Indigenous populations

Indigenous‐led models of health care are a fundamental requirement to creating innovative PHC solutions that are both effective and sustainable.^6^ Co‐design principles can thus provide pathways for potential engagement of Indigenous consumers and stakeholders in research that leads to effective health care and improved health outcomes.^11^ To develop successful solutions, all aspects of the co‐design process need to include Indigenous leadership and governance, as well as consumer participation.^6^, ^12^, ^13^, ^14^ Consequently, developing digital models of health care for Indigenous populations requires a systematic, culturally appropriate, and co‐designed approach to ensure widespread adoption and long‐term sustainability. However, the potential benefits of digital health solutions for Indigenous communities, particularly those in rural and remote areas with limited access to health care and digital technology, remain largely unexplored.

### 1.2 Goondir's virtual health services

This study partners with the Goondir Health Services (Goondir), which is an Aboriginal Community Controlled Health Organisation (ACCHO) servicing six regional and rural locations across Southeast Queensland. Goondir operates four clinics: Dalby (classified under Modified Monash Model [MM]^15^ as MM4), Oakey (MM5), St. George (MM6), and Chinchilla (MM4), servicing a population of more than 5000 Indigenous consumers. Amongst active clients, more than 63% have a diagnosed chronic disease according to current administrative data held by Goondir. In 2020, just prior to the COVID‐19 pandemic, Goondir implemented a proactive approach to support rural and remote consumers living with chronic diseases, through the implementation of a Virtual Health Service (VHS). The VHS framework utilises a genuine Indigenous‐led approach based on the Gayaa Dhuwi (*proud spirit*) declaration and ensures Indigenous peoples' cultural authority, including connection to Country and culture; spirituality, ancestral ties; resilience and kinship; and community leadership and governance.

Goondir's VHS model includes five different blue‐tooth enabled RPM devices: a Samsung internet‐connected tablet or a phone app, a P3 pulse oximeter, Bg5 blood glucose monitor, blood pressure monitor, and a wireless weighing scale. Eligible adult consumers who meet certain intake criteria as determined by a team of health professionals including General Practitioners, receive a VHS kit to use in their own homes, are provided training to take measurements, and are encouraged to take daily readings of all their health vitals. These devices are connected to a digital platform where patient health records are monitored on a clinical dashboard, accessible by Goondir staff in any of their clinics. Abnormal health vitals are flagged to relevant clinicians by health coaches for further follow‐up and assessment through a secure video call, using the consumer's inter‐connected tablet or phone applications. To ensure continuous quality improvement, ‘health coaches’ regularly communicate with VHS consumers via phone calls and in‐person visits, enabling safe, continuing, and effective fit‐for‐purpose health services. Health coaches are dedicated Aboriginal Health Workers who have been specifically trained as part of the VHS model.

The VHS model has grown to include consumers across a wider service region, in partnership with several other ACCHOs. These include the Nukal Murra Alliance across rural and remote Western Queensland through Charleville (MM7), the Western Areas Aboriginal and Torres Strait Islanders Community Health service (CWAATSICH) (Charleville region) (MM7), Gidgee Healing (Mt Isa region) (MM7), the Cunnamulla Aboriginal Corporation for Health (CACH) (Cunnamulla region) (MM7), and the Mulungu Aboriginal Corporation Medical centre in North Queensland servicing the Mareeba (MM4) & Atherton areas (MM4).

### 1.3 The ID‐INSPIRED project

Understanding the need for evaluation through research, Goondir Health Services (FL) partnered with researchers (BN, SKC, MM) from The University of Queensland in 2022. This partnership led to the co‐design of the Innovative Digital‐IndigeNouS PrImaRy hEalthcare Delivery (ID‐INSPIRED) project. Through ID‐INSPIRED, improvements leading to the long‐term sustainability, effectiveness, digital improvements, and cost‐efficiency of the VHS are being conducted. This manuscript aims to describe the co‐design processes of the VHS model. Furthermore, it explores the experiences of VHS consumers.

## 2 CO‐DESIGN METHODS

### 2.1 Indigenous governance and engagement

Driven by existing engagement, an Indigenous Governance Committee (IGC) was established, consisting of consumers and non‐consumers, service providers, health coaches, and a researcher (BN) to provide oversight of the study across all stages. Community members and consumers were invited via word‐of‐mouth invitation, including agreeing to the terms of reference. Consumers were key members of this committee to enable lived experience and end‐user feedback as part of the co‐design process. Community members, who are not consumers, are also essential members of this committee, to provide an unbiased perspective. The IGC maintains all codes of conduct and ensures that cultural and clinical practices are implemented throughout the study.

### 2.2 Study design

We employed decolonising methodologies^16^, ^17^ to co‐design the ID‐INSPIRED model, as well as an Indigenous consensus method.^18^ This method enables Indigenous values and culturally relevant processes to take place that provide a focus on consumer and community voices. It also incorporates Community‐Based Participatory Research (CBPR) principles.^19^, ^20^ CBPR is a partnership approach to research that equitably involves community members and researchers in all aspects of the research process and in which all partners contribute expertise and share decision making and ownership. The key Indigenous research principles of co‐design were maintained throughout the study.^11^ Further, a qualitative interpretive description approach^21^ was used as an overall framework to guide the research. This methodology is a flexible, non‐prescriptive qualitative approach designed for the development of new knowledge, with fundamental principles that are compatible with, and complementary to, both participatory and decolonising research.^22^ Furthermore, it allows the researcher and participants to co‐create or co‐construct understandings, thus enabling co‐design.^23^

### 2.3 Participants and recruitment

Eligible participants were all Goondir clients residing in MM3‐7^15^ communities, were above 18 years of age and were able to provide informed consent. Participants were invited through word‐of‐mouth at frequent community consultation engagement events such as VHS information sessions held across multiple communities within MM3‐7 regions, though only one community contributed participants to the study.

### 2.4 Data collection

**FIGURE 1:**
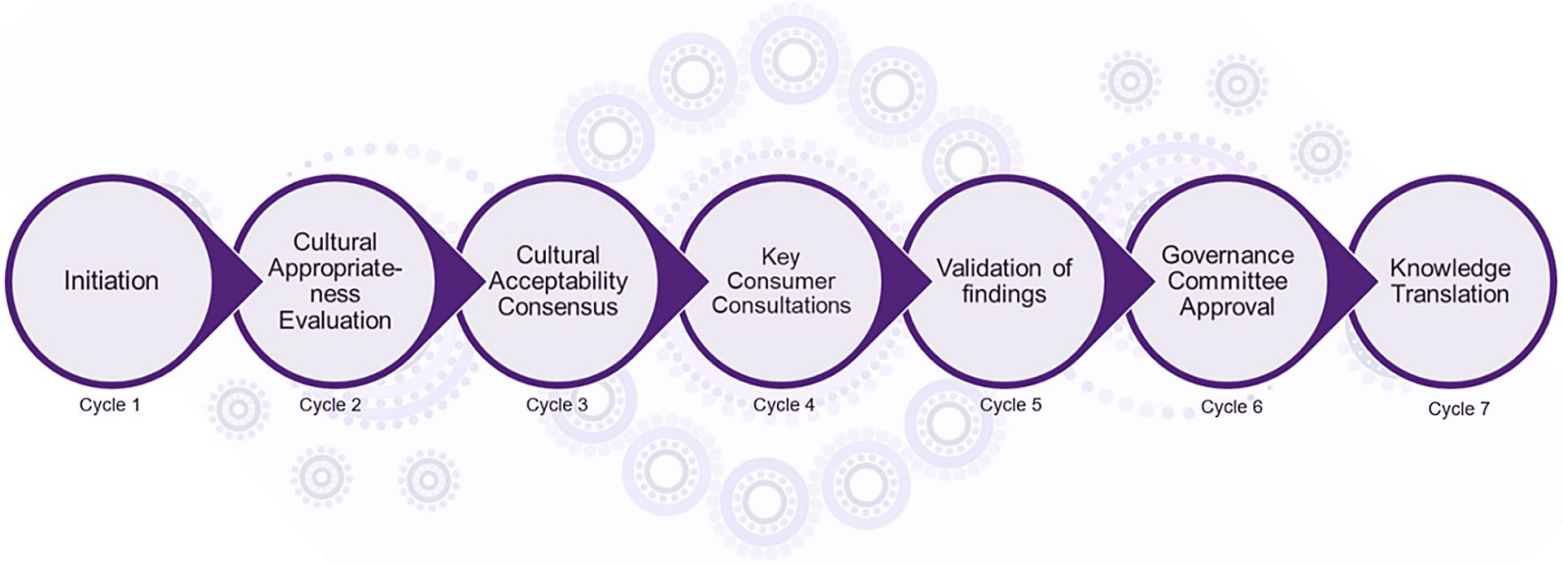
The adapted Indigenous consensus method.^18^

The Indigenous knowledge circle consensus method, first described by Maar et al. (2010),^18^ involves 7 cycles of continuous consultation based on qualitative research methods that are well‐aligned with acceptable Indigenous research methods.^12^, ^24^ These cycles were modified for the purposes of this study as described below (Figure 1). (BN) a non‐Indigenous, female, health researcher with more than 10 years of experience conducting Indigenous health research, facilitated data collection in close collaboration with the IGC.

- *Cycle 1: Initiation*: A guided face‐to‐face discussion with the IGC and consumers was conducted to identify parameters to define an enhanced virtual model of care based on the VHS model. Parameters were discussed and modified until a consensus was reached.
- *Cycle 2: Cultural evaluation*: A yarning session was conducted to discuss the co‐design of agreed parameters from Cycle 1. Key outcomes were collated and presented to the IGC for review and endorsement.
- *Cycle 3: Cultural acceptability consensus*: The IGC reviewed the outcomes from Cycle 2 and provided feedback on the practical co‐design and implementation modifications that may be required to ensure the model is culturally acceptable and useful in a clinical setting.
- *Cycle 4: Consumer consultations*: After necessary modifications were made to the model, consumers and non‐consumers were invited to two focused yarning sessions. Thematic analysis of the notes from the yarning sessions was conducted, and key themes were generated. The guiding questions were co‐created with members of the IGC.
- *Cycle 5: Validation of findings in the context of current literature*: A draft report was prepared for the IGC which included outcomes based on the qualitative analysis of the data collected from Cycle 4. The report was discussed in the context of current literature that defines digital PHC service models for Indigenous populations.
- *Cycle 6: Validation with the Governance Committee*: The draft report from Cycle 5 was presented to the IGC for further discussion, modification, and finalisation until the committee reached a consensus on the final model.
- *Cycle 7: Dissemination and knowledge translation*: The final report was then modified into an academic publication (this manuscript). Accordingly, this manuscript was reviewed by the IGC and submitted after their approval.

### 2.5 Data analysis

Thematic analysis was conducted using a hybrid of inductive and deductive coding of anonymised transcripts.^25^ The initial analysis involved independent coding of data by a research team member (BN) using NVivo, with verification by another member (WM). Once codes were developed and agreed they were reviewed by a third team member (PM). Common themes were developed following data interpretation and analysis. The generated themes were presented to the IGC for validation through an Indigenous interpretive lens enabling meaningful data insights. The IGC provided a final review and approval, prior to any dissemination of outcomes to academic journals or conferences.

### 2.6 Trustworthiness

A number of measures were used in this study to ensure trustworthiness.^26^ Researcher triangulation was ensured by three qualitative experts (BN, WM, PM) undertaking the data analysis through regular meetings for peer checking. Dependability was ensured through regular discussions and the use of coding with the IGC. The researchers ensured confirmability by maintaining reflexivity throughout the study processes and used the Indigenous knowledge circle consensus method, incorporating different perspectives, assumptions, and biases.

### 2.7 Ethics approval

The study was approved by the University of Queensland Human Research Ethics Committee (2022/HE002522).

## 3 RESULTS

The Indigenous knowledge circle consensus method commenced with the establishment of the IGC in September 2023. Cycle 1 of the methodology was conducted in August 2023 with IGC members, three consumers and one non‐consumer. Key parameters generated from the IGC in Cycle 1 were put forward to guide future cycles. Subsequent Cycles 2–4 were conducted over a period of 4 months (September–December 2023), with the Cycle 4 focused yarning sessions conducted in February 2024. A total of 14 consumers and one non‐consumer participated in the focus group yarning sessions. All participants were from a single MM4 community. A descriptive report was prepared and presented to the IGC for review and approval. This manuscript presents the key themes identified that are required for a culturally acceptable and co‐designed digital model of care. Some consumers had a dual role where they were also a health coach. To protect participant anonymity and privacy, all quotes below have been presented as consumers (labelled C).

### 3.1 Themes

Two main themes were developed: (1) personalised approaches to autonomous care using digital technology and (2) person‐centred, culturally appropriate healthcare elements within a VHS model.

#### 3.1.1 Personalised approaches to autonomous care using digital technology

Within this theme, two sub‐themes were developed on the benefits and challenges of technology and the integration of culturally inclusive healthcare elements. Each sub‐theme portrays different aspects of using digital technology as a mechanism to provide independent and sovereign health care for chronic disease patients. This personalised approach using digital technology was seen as a positive and empowering means to providing essential health care for consumers.

##### The benefits and challenges of using culturally responsive technology

Many participants discussed the use of digital technology through a cultural lens. They explained that digital technology provides a way for consumers to feel empowered to look after their own health. They noted that this is a culturally responsive approach to personalised health care that is respectful, relevant to health beliefs, health practices, cultures, and needs of Indigenous consumer patient populations and communities:

So, for the patient, it's for themselves, they can look at their readings and go oh, you know, maybe I should make a doctor's appointment and it then it gives them that direction on where they're going… it gives you as a patient an overview of where you are with your health as well. And all of this is something that fits well with our culture and ways of doing things. C01

Consumers of the VHS spoke about this digital model of care providing more constant access to health care, as one participant explained:

Like for me, I now don't see my doctor every week. I may see him every three months or whatever, but he has that record of what my blood pressure is doing or what my weight's doing, that type of stuff around medication and ongoing issues and what not. So, I think it provides more constant access. C04

The use of digital devices also allows for efficient patient monitoring, and in some situations, has even enabled prompt patient care, ‘saving lives’ of high‐risk chronic disease patients. One participant said:

As we say to everyone, the devices are not lifesaving, they are monitoring data collection for you and for your doctor to see a pattern or see how things are going for you. But in saying that, they have saved lives, just the two that I know of. Well I was looking at somebody's results in and gave him a call. His pulse was extremely high, like 120. He was on 124. I rang him. Said, “How you doing?” He said “I'm not doing good. I'm glad you called.” I said “What have you done?” And he said “I can't walk. I'm vomiting. I can't stand. My head is spinning.” I said “have to get the ambulance,” he said “Yeah.” So, we got the ambulance. With him, the ambulance got him to the doctor… He rang back the next day, saying he'd taken the medication wrong. So just by getting through straight away and that's that app, you see that integration. C01

Alongside the many benefits, participants also highlighted suggestions for improvement and issues with the technology that they had experienced. A well‐being element within a digital model of care was discussed as a potential inclusion to support consumers' mental health, as one participant explained:

Some things I would like to see added on there actually, one being that on this day I'm feeling like ****. I've gone through a divorce or I've got stuff happening. They're just a little “How are you feeling today?” Just a little click of a button. That's how I'm feeling today. I'm good. I'm no good. I'm cranky. Because sometimes those things are real. And they affect your blood pressure. If you're feeling really down and you're still taking your readings. C07

Some participants experienced technological challenges. They noted:

They still need to iron out a lot of incorrect readings coming through and we're not sure whether it's the machine that they have. Or it's them actually using the machine, putting the cap on back [incorrectly]. So you know, we need to sort out what's an actual incorrect reading to an actual correct reading, you know. C01

It doesn't connect and then you'll be halfway through doing your readings and it'll just shut down and you gotta press the thing again and start all over again. Sometimes I don't have time for that. C07

However, the option to choose between an app or a tablet in the VHS kit, based on the participant's preference, was seen as beneficial:

But I mean you've got both options available still. The app might work better just because of the way it functions and [is] so much more convenient, but for others there's still the option of having the tablet. C03

With the app, it's brilliant. I like it. It saves me time. C02

The features of the app provided significant benefits as a resource during medical appointments, and a way for consumers to save money and time:

The other thing about the app is when I go to a specialist like the cardio or the endo, I can just do it on my phone and all those numbers are there for all my readings, so they can actually see every day what my BSL is. My weight is, my you know, my blood pressure or whatever, and it's all there. So, that's a massive bonus because the doctors don't have to send pages and pages and pages of paper on the referral. CO3

It saves so much time and I don't need to visit the clinic so that's a good thing. C09

##### The integration of culturally inclusive, tailored healthcare elements

Participants discussed the integration of elements that could make the digital technology more culturally inclusive and user friendly, potentially increasing its uptake and use:

Because digital technology is for everybody. So, this is where it comes in to being appropriate by adding… that Aboriginal touch to it, like icons and people who wanna make it personal with their family and stuff. Like that is what matters to them then that would be culturally appropriate and make more people use it. C04

Incorporating culturally relevant design elements were perceived as a means to ‘open [VHS] up’ and make it ‘not so clinical’ and ‘more welcoming’ (C07). Australian Indigenous populations often experience a pervasive fear of accessing and utilising health care,^27^ consequently non‐clinical and welcoming healthcare settings are more culturally appropriate. The word choice of welcoming, that is, ‘welcome’ has a lot of Indigenous connotations. Equally, minimising the ‘clinical’ has a lot of historical significance given the removals of children by healthcare practitioners in the past.

Hopefully, when they open it up and it's not so clinical. You know it just makes it more welcoming when you give it to them. C07

Other participants noted improvements in the design that could potentially improve the likelihood of app usage.

And I think the other thing too, that might actually help is where you turn on the screen and just the blank thing. And we're talking about design and whatnot, having a daily quote, an inspirational quote, you know just something they look forward to reading it every day. It's something new. It might just give them the incentive to actually turn the tablet on to see what's on there. C09

#### 3.1.2 Person‐centred, culturally appropriate healthcare elements within a VHS

The second overarching theme focused on various parts of the VHS that make it culturally appropriate and encourages its uptake and use by consumers. Three sub‐themes were developed within this category on the vital role of health coaches, importance of community connections and enabling holistic personalised healthcare access.

##### The vital role of health coaches

The vital role of the health coach, and the type of person and skills required to undertake this role, were identified as significant factors that make a VHS designed for Indigenous patients' function successfully. Participants said:

And I think those health coach positions is what makes this program culturally appropriate. Yes. Because if we had white [registered nurses]. Or people who don't have connections to the community. Yeah, it wouldn't work so well because people wouldn't answer their calls. They wouldn't open up the way they do, and we wouldn't get to the bottom of a lot of the stuff that's going on. C010

It's something you're adding to your morning routine that takes quite a few little steps to get going…being a little bit overwhelmed is enough to push it away and go ‘I'll do it again later’ and nine times out of 10, you're not going to. And a lot of our people won't reach out for help and go…Yeah, and that's the beauty of our new support officers now in the community where they can help us. C013

The language used by health coaches was discussed as a vital element of sustaining the progress of the VHS.

It's also the language. The way you guys speak when you come into the home, you're not talking like a white [registered nurse]. Yeah, I talked to [name] about it today – about code switching. Which we learn to do but don't realise we can do it. So, you can sit in a conference and talk your work stuff and then go to a patient's house and you would talk just like we talk in community. C04

##### The importance of having community connections as a health coach

Having health coaches that were local to the consumer's community helped them establish connections with VHS clients, allowing them to provide ongoing support and health services to them. One participant said:

And I think sometimes too, [it's] the great connection in the community they have [health coaches]. So, she knows people and she knows past activities and people and events, so they have a big connection, so that's important. I think that is a lot to do with that. I've got one lady and she'll only let [health coach] in her house, but I like that. I think that's really important as well. I think, she feels safe and heard and being listened to. Yeah, it's part of what I think makes this program work. C014

Maintaining regular contact with consumers, the health coaches are regarded as an important element of the VHS due to the connections they maintain and nurture within their communities:

The really positive thing I like about VHS, like besides helping with the chronic diseases, it's the contact. Yeah, it's the phone calls. Yeah, that's what I really like the connections I hear. Like, I rang somebody the other day and they said, “are you ringing me this morning [because] my sugars were high? and I was waiting [for your call]”. C011

Its excellent for our people not just about the blood sugars, we can talk to them about other things, so I love to have those community connections. C02

##### Enabling holistic personalised healthcare service access

Speaking with consumers of the value of the VHS and whether it enables access to holistic and personalised health care, participants indicated how this model of care has empowered them to take ownership of their own health:

I just think that it has made me take control of my chronic disease myself, not relying on the doctors and medication but made me realise if I don't do anything it's going to get worse not better. C09

Participants valued the personalised approaches taken by health coaches. Participants described how health coaches provide holistic care, including ensuring that each patient receives tailored health care which is suited to their needs. One participant said:

I think for some people there actually are a lot of steps… I think it's just too many steps for some people, and that's why I've said if giving it to someone who's not sure with the new devices, let's just give them one. Let's just do one device at a time. C013

## 4 DISCUSSION

Using a modified seven‐cycle Indigenous knowledge circle consensus method,^18^ this study generated two themes describing the requirements of a culturally acceptable and co‐designed digital model of care. Namely, personalised approaches to autonomous care using digital technology and person‐centred culturally appropriate healthcare elements within a VHS model. Five sub‐themes further describe these requirements and demonstrate the potential for personalised digital health technology to bridge geographical and cultural barriers for the management of chronic disease for Indigenous Australian populations. The study highlights how VHS technology offers promising benefits, including improved access to prompt quality health care, culturally safe and respectful health provision, enhancement of consumers' self‐management of chronic diseases, and the importance of having access to local Indigenous community‐based health service provider support.

This study identified a particular preference of VHS consumers for having access to personalised, autonomous control over the technology as part of the VHS model. This enabled consumers to benefit from cost‐savings, reduced travel burden, and improved continuity of health care. Other studies have previously identified that telehealth, in particular, provides similar benefits.^7^, ^8^ This study also identified how virtual models of care can contribute to providing streamlined access to specialist services. Consumers in this study reported the ease of health information availability during specialists' visits through the VHS phone apps, the value of monitoring health vitals regularly, and the importance of having control over their own health. These findings indicate that the VHS model is successfully improving the patient's health journey. Likewise, other studies have reported how telehealth in particular may improve consumer outcomes, and access to specialist services.^8^ Despite the technological challenges, the VHS technology is able to ensure safety, and equitable access to health care, thereby empowering consumers. Although consumers of the VHS model identified some barriers to usage and opportunities for further improvement, most consumers were supportive of the model and highlighted cost and time saving as important benefits. The perceived effectiveness and utility of the software application, whether accessed through the tablet or the phone apps, was deemed as an integrated and valuable part of the model of care by consumers. However, the medical and clinical attributes of the VHS software application and its Western designs did not generate any warmth from the participants. They expressed a desire to see the application overlaid with Indigenous designs or modified to allow background images of participants' relatives or pets. This may indicate a desire to include the VHS application within their existing relationship networks and use it to remind them of their responsibility to manage their health conditions for others.

Relationality is a fundamental aspect of Indigenous culture which is about unity, kinship and responsibility.^28^ Relationality emphasises the connections between all things^28^ and is reflected in the themes and sub‐themes generated from Indigenous participants' perspectives. The importance of relationality was therefore evident in participant views on the design and layout of the VHS application. The individualisation of the application allows the participant to see or feel themselves represented as an individual within the collective (their community, family, Country). For instance, a participant may add family photos in the application. A non‐Indigenous participant may interpret this choice as ‘personalisation’ with the aim of making the app more about them: ‘this is my app and my family’. An Indigenous viewpoint may instead view this personalisation as ‘this is the family I belong to’ with personalisation thereby serving as a reminder that ‘I look after myself for them’. Though the difference is subtle from a western perspective, the internal emphasis on duty to others (Relationality: unity, kinship and responsibility) is the important distinction. We would hypothesise that if the app is used by a non‐Indigenous population the lack of personalisation would only be a minor problem as participants would be largely pre‐conditioned to be interested in the self (individualism). On the other hand, our research suggests that a lack of personalisation for an Indigenous user group may restrain their ability to view use of the app as something they do to look after themselves for their community, allowing them to continue to contribute to it in the future (relationality). More broadly, personalisation of digital interventions, such as the VHS program, may serve as a mirror, allowing users (largely Elders, Aunties, Uncles) to perceive themselves as part of the community and Country (unity). Their responsibility, generally speaking, is then to use digital intervention, live longer, and help the next generation.

The present study also identified the importance of utilising culturally appropriate, person‐centred, holistic healthcare elements as part of a VHS model. Another study evaluating the cultural appropriateness of health care using telehealth similarly identified the importance of Indigenous health workers presence in facilitating culturally appropriate service provision.^7^ Providing advocacy, assistance and a supportive environment, the study identified the value of telehealth services delivered in a safe and respectful way.^7^ Patient outcomes may thus be underpinned by the unique design aspects of the VHS model. The VHS model was designed and led by Indigenous peoples and is therefore underpinned by a relationality. In this VHS model, the inclusion of local Indigenous health coaches is seen as crucial to its success. Though the VHS uses digital services and data readings are collected remotely and independently by consumers, regular check‐ins with health coaches are a strong feature of the model. The health coaches assist in taking measurements which consumers might find ‘tricky’, provide technical support (e.g. device updates, connectivity issues), and organise other care unrelated to the VHS (e.g. specialist appointments). Because they belong to the local community, the connections established by the health coaches with the consumers also facilitates general health and well‐being check‐ins, deliver support when needed, and provides connection to community and culture. The importance of connection to healthcare providers of the same culture is also reported in other Indigenous literature.^6^, ^29^, ^30^ The role of health coaches is perhaps more unique though, given their ability to provide clinical level care, outreach care, home visits, and their oversight of consumers' daily VHS health data and subsequent follow‐up. Furthermore, participants in this study emphasised the importance of health coaches being Indigenous people from the local community: they placed a high value on the health coaches' existing social networks and respected their past and ongoing contributions to the local communities.

Previous research in Australia has identified that virtual models of care can improve access to health care; however, there remains limited evidence highlighting the effectiveness, access, equity, utility, safety, and quality of these technologies.^5^ There is also very limited evidence of the use of virtual PHC models within Indigenous populations, with a recent rapid review identifying only seven studies from Australia ranging from between 2012 and 2021.^6^ To the best of our knowledge, there were no studies identified that investigated the cultural appropriateness of integrated VHS models of PHC for Indigenous populations, beyond telehealth services. For the first time, this study has addressed gaps in evidence by providing a comprehensive insight into the enablers and barriers of VHS models of PHC delivery, their cultural appropriateness for Indigenous consumers, as well as factors that can enhance future digital health service provision. Improving access to health care using digital health technology is a potential approach for meeting the needs of Indigenous populations, especially those residing in regional, rural, or remote communities.

### 4.1 Strengths and limitations

The strength of this study lies in the co‐design component, enabling participant voices to be heard clearly. Another advantage is the use of complementary frameworks and methodologies, such as decolonising methodologies, CBPR principles and the qualitative interpretive‐descriptive approach. The broad range of chronic conditions monitored within the VHS program also adds to the generalisability of the results of this study. The primary limitation of this study was the limited geographical and social diversity of participants. While sample size was sufficient to conduct the study, the generalisability of the findings may be limited as all participants were from the same community. The generalisability of the outcomes to other remote communities may also be limited, since all participants were from an MM4 community. Consideration was given to recruiting VHS participants from other communities; however, facilitating travel, engagement, and consultation with participants was restricted at the time of the study due to the availability of the IGC members.

## 5 CONCLUSION

This study identifies the importance of personalised approaches to autonomous health care using digital technology, and person‐centred, culturally appropriate healthcare elements within a virtual healthcare service model based on Indigenous consumer perspectives. The study highlights the importance of such technology to bridge geographical and cultural barriers to improve chronic disease management for Indigenous Australians with chronic disease and living in rural areas.

Our findings suggest Indigenous digital healthcare models should be designed using relationality approaches. They must reflect and truly be a part of the Indigenous communities that they seek to serve. Further research is necessary to develop and evaluate sustainable digital PHC models that bridge these divides and harness the full potential of personal digital health technology for improved health management in Indigenous populations', while ensuring that the unique needs and preferences of Indigenous communities are being met. Moreover, while consumer perspectives are critical to assessing a digital health service, further research of health outcomes will also be essential to determine its overall impact for Indigenous health service consumers.

## AUTHOR CONTRIBUTIONS

BFN: contributed to the conception, design, data acquisition, analysis, and interpretation, drafting and critically reviewing the manuscript, and providing final approval for dissemination. WM: data acquisition, analysis, and interpretation, drafting and critically reviewing the manuscript, and providing final approval for dissemination. FL: contributed to the conception, interpretation, critical review of the manuscript' and providing final approval for dissemination. PM: contributed to the conception, interpretation, critical review of the manuscript' and providing final approval for dissemination. KA: contributed to the conception, interpretation, critical review of the manuscript and providing final approval for dissemination. KW: contributed to the conception, interpretation, critical review of the manuscript and providing final approval for dissemination. MM: contributed to the conception, interpretation, critical review of the manuscript' and providing final approval for dissemination. SKC: contributed to the conception, design, critically reviewing the manuscript, and providing final approval for dissemination.

## FUNDING INFORMATION

This project is funded by a National Health and Medical Research Council (NHMRC) 2021 Medical Research Future Fund (MRFF) in Primary Health Care Digital Innovations (APP2023585).

## CONFLICT OF INTEREST STATEMENT

The authors declare no conflict of interest.

## ETHICS STATEMENT

The study was approved by the University of Queensland Human Research Ethics Committee (2022/HE002522).

## Data Availability

The data that support the findings of this study are available from the corresponding author upon reasonable request.
